# Extracorporeal Shockwave Therapy for Patients with Chronic Achilles Tendinopathy in Long or Short Course

**DOI:** 10.1155/2020/7525096

**Published:** 2020-08-11

**Authors:** BaiLi Yan, Yuan Wan, Hong Zhang, MengTing Pan, Cheng Zhou

**Affiliations:** ^1^Department of Pain Management, Wuhan No.1 Hospital, Wuhan, China; ^2^Department of Hepatobiliary Surgery, Wuhan No.1 Hospital, Wuhan, China

## Abstract

**Methods:**

The data of 66 patients with CAT received ESWT was reviewed. According to the disease courses, those cases were allocated to short-term group (ST group, symptom duration 3-6 months) and long-term group (LT group, symptom duration >6 months). Propensity scores match (PSM) method was conducted to eliminate the confound factors in baseline features including gender, sport history, sides, type of CAT, BMI (body mass index), age, and scores evaluated by AOFAS (American Orthopedic Foot and Ankle Society) and VAS (Visual Analogue Scale) before ESWT. After balancing the features between ST and LT group, postinterventional VAS, AOFAS, and rate of Likert satisfaction scale at the 3rd month after first ESWT was statistically analyzed.

**Results:**

Among the baseline features in ST and LT group, gender (female ratio, 44.4% vs 71.4%, *p* = 0.041) and BMI (23.26 ± 2.15 vs 24.63 ± 2.41, *p* = 0.024) were identified as confound factors. After elimination of biased features with PSM, 3 months after first ESWT, AOFAS and VAS in both groups are significantly improved, when compared with their scores at baseline (*p* < 0.01). Moreover, at postintervention month 3 (PIM3), AOFAS in ST group is significantly higher than LT group (85.08 ± 9.83 vs 76.76 ± 9.85, *t* = 76.76 ± 9.85, *p* = 0.019), and the rate of Likert satisfaction in ST group is better than LT group; although, it did not reach but close to significant level (70.6% vs 47.1%, *χ*^2^ = 1.943, *p* = 0.163). However, there is no statistical difference of VAS scores between two groups after ESWT (1.96 ± 0.98 vs 2.24 ± 1.29, *t* = 0.703, *p* = 0.487).

**Conclusions:**

ESWT could effectively relieve pain and improve function of hind foot in patients with chronic Achilles tendinopathy, and especially, it could offer better benefit on functional improvement in patients with short duration of CAT symptom.

## 1. Introduction

Chronic Achilles tendinopathy (CAT) is a common pain disease and prevails in athletes, middle-aged male runners, and sedentary population [1]. In China, with the enhancement of awareness of sports and health, the number of physical activity participants is rising; according to the report from Chen et al. [2], the percentage of daily exercisers in Chinese urban resident has reached 19.57%, unfortunately, of which, CAT occurs more frequently in running population, for about 5-18% [3, 4] of incidence. Typical symptom of CAT composes of Achilles pain, swell, and functional limitation. CAT is complicated in its pathogenesis, which is not fully elucidated yet. Conservative therapies, such as immobilization, nonsteroid anti-inflammation agents, Laser therapy, hyaluronan (HA) or platelet-rich plasma injections, and eccentric exercises are all regarded as treatments in the first line.

However, the extracorporeal shockwave therapy (ESWT), first proposed in management of CAT by Furia [5], has advantages of safety, minimal invasive, versatile, economic, which makes it become gradually increasing of application in clinical practice, and an important supplement for conservative therapy as well. However, in Stania et al.'s [6] meta report, by analyzing of 22 clinical trials, he believed that because of complexity of biologic reaction results from CAT, great diversity among different ESWT algorithms, and lack of objective parameters for outcome measurements, the value of ESWT in CAT therapy is not fully evaluated. In addition, there is also an opinion that indicates longer duration of CAT directly correlated to insufficient therapeutic effects offered by ESWT [7], but to the best of our knowledge, there is no literature concerning the relationship between the CAT duration and ESWT efficacy. For aforementioned reasons, we tried to explore if early ESWT intervention is a better approach for the recovery of patient with Achilles tendinopathy. The data of patients received ESWT because of CAT in our institute was collected and analyzed, retrospectively, and reported as follows:

## 2. Material and Methods

The medical records of patients suffered from CAT and treated with ESWT at outpatient or inpatient of pain management department in our institute, from Dec. 2017 to Dec. 2019, were collected retrospectively. There are 66 cases in total. Diagnostic standard of Achilles tendinopathy is defined as having pain and tenderness located on unilateral or bilateral Achilles area. Inclusion criterion is the patients clinically diagnosed as CAT with or without radiology evidence, which had a symptom persisted at least 3 months. Exclusion criteria were settled as patients who administrated with nonsteroid anti-inflammation agent within one week before ESWT or received steroid therapy or previous ESWT within 3 months before ESWT, or concomitant with anatomic foot malformation, fracture, or operation history of foot and heel. Ethical approval is not needed because of the retrospective natural in our study.

### 2.1. Case Grouping

According to the duration of CAT symptom, 66 patients were divided into long-term group (LT) for 21 cases, symptom persisting longer than 6 months; short-term group (ST) for 45 cases, duration from 3 to 6 months.

### 2.2. Type of CAT

Insertional type defined as pain located at 2 cm within the insertion of tendon into the calcaneus. Noninsertional type is defined as pain located 2-6 cm proximal to calcaneal insertion point [8].

### 2.3. Functional Parameter of Foot and Ankle

Scores system developed by American Orthopedic Foot and Ankle Society (AOFAS) was applied in our study [9]. 100 points were designed in AOFAS system in total, in which 40 points stand for pain severity, 50 points present function state, and 10 points for alignment.

### 2.4. Pain Parameter

Visual Analogue Scale (VAS) was performed [10]. From 0 to 10 points (10 points = serious pain, 0 = painless), patients were asked to decide the scores based on their subjective perception of pain severity.

### 2.5. Satisfactory Rate Parameter

6-points Likert satisfaction scale was used in our study (Table 1). Satisfaction was defined as 1-4 points fed back by patients, unsatisfaction was regarded as scores from 5 to 6 points, and rate was calculated for further analyze.

### 2.6. ESWT Procedure

Equipment we used in our study is a radial extracorporeal shockwave instrument (Swiss DolorClast Smart20, EMS Elctro Medical Systems, Munich, Germany). In each therapy episode, frequency was set as 4 Hz or 8 Hz and 2000 pulse, and 0.096-0.137 mJ/mm^2^ was given. Patients took on prone position, and the most tenderness area complained by patients was treated as target region. ESWT manipulators were specialized physiotherapists in our department. During procedures, therapists exert pressure depending on patient's maximal tolerance of pain. 3-5 sessions of ESWTs were given at weekly interval without any additional exercises. Except for diclofenac diethylamine emulgel (Novartis China, Beijing) was used as lubricator before intervention; no other analgesic agent was given. Patients were educated to avoid vigorous physical activities during treatment session.

### 2.7. Follow-Ups and Parameters

**Figure 1:**
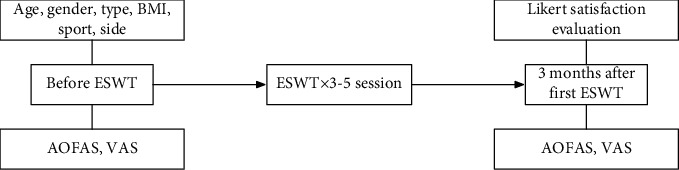
Flow-chart of parameter evaluation and follow-up.

In all of 66 cases, the data regarding gender, sport history, sides (superiority or nonsuperiority), type (insertion or no insertion), BMI (body mass index), age, AOFAS scores, and VAS scores evaluated before first ESWT were collected as baseline features. Patients were routinely followed up at outpatient at the 3rd month after the first ESWT, the scores of AOFAS, VAS, and Likert satisfaction were reevaluated, which were collected as outcome parameters for statistical analysis (see Figure 1). Adverse events after ESWT was also recorded.

### 2.8. Statistical Analysis

Continuous variables were tested with Kolmogorov-Smirnov for normality of distribution first and then compared between LT and ST groups by Student's *t*-test. The variance of categorical variables was determined with chi-square test. To minimize the bias between LT and ST groups at the baseline, we performed 1 : 1 propensity score match (PSM) with the nearest neighbor matching algorithm and set parameter of match tolerance as 0.1. After match, the variance derived from intergroups and intragroups was further analyzed with two tails Student's *t*-test in quantity data, and by chi-square or Fisher's exact test in quality data. We used SPSS 24.0 (SPSS Inc., Chicago, Illinois, USA) for all statistical analysis, and *p* < 0.05 suggested a statistically significant difference.

## 3. Results

First, we compared factors including gender, sport history, sides, type, BMI, age, and preinterventional AOFAS and VAS scores between ST and LT groups, to identify the bias characteristics at baseline (Table 2). Among enrolled 66 cases of CAT, 68.18% was in ST group (*n* = 45), and 31.81% in LT group (*n* = 21).

Based on baseline characteristics before match, regarding to gender ratio, CAT influenced significantly more female patients in LT group than ST group (71.4% vs 44.4%, *p* < 0.05); meanwhile, BMI was also significantly higher in LT group (24.63 ± 2.41 vs 23.26 ± 2.15, *p* < 0.05). In terms of age factor, patients in LT group is elder when compared with ST group; although, the difference did not reach, but very close to significant level (60.80 ± 8.74 vs 56.77 ± 8.68, *p* = 0.084). The variances among other features are all insignificant.

After balanced with 1 : 1 propensity score matching, the data of 34 patients was preserved, and 17 cases for each group. 8 baseline characteristics and 3 outcome parameters, including AOFAS, VAS, and Likert satisfaction rate at the 3rd month after first ESWT, were further compared and analyzed. See Table 3.

After matching, the female ratio at baseline changed to 64% in ST group and 76.5% in LT group; no significant difference was identified anymore, *p* = 0.45. Moreover, the bias of BMI between two groups is no longer existing, when compared ST group (23.96 ± 1.93) again with LT group (24.11 ± 2.25), *p* = 0.831. Between two groups, the balance of age factor remains unchanged (59.91 ± 9.62 vs. 61.34 ± 9.33, *p* = 0.662).

**Figure 2:**
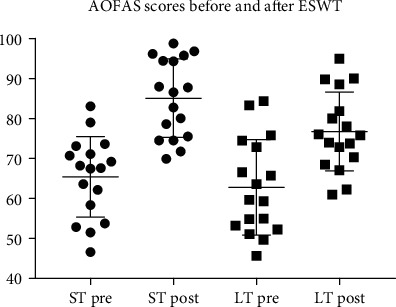
AOFAS scores before and after ESWT.

**Figure 3:**
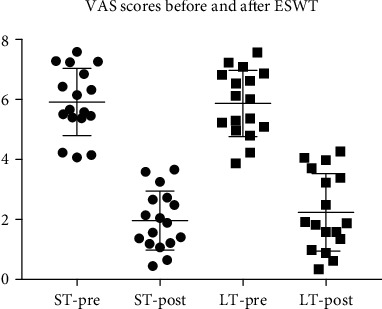
VAS scores before and after ESWT.

After PSM, we compared AOFAS and VAS scores of both groups with their baseline counterpart and also check the difference on postinterventional AOFAS, VAS, and Likert satisfaction rate between ST and LT group (Figures 2 and 3).

After PSM, at the 3rd month after first ESWT, compared AOFAS scores between two groups, ST group (85.08 ± 9.83) is significantly higher than LT group (76.76 ± 9.85), and *p* value was 0.019; however, there is no significant difference of VAS scores was observed (1.96 ± 0.98 vs 2.24 ± 1.29, *p* = 0.487) between two groups. Compared with baseline, postinterventional AOFAS scores in ST group is significantly increased (65.43 ± 10.05 vs 85.08 ± 9.83, *p* < 0.01), and VAS scores reduced significantly (5.92 ± 1.12 vs 1.96 ± 0.98, *p* < 0.01). The similar tendency of peri-interventional changes of AOFAS (from 62.80 ± 11.90 to 76.76 ± 9.85, *p* < 0.01) and VAS (from 5.87 ± 1.10 to 2.24 ± 1.29, *p* < 0.01) was seen in LT group.

**Figure 4:**
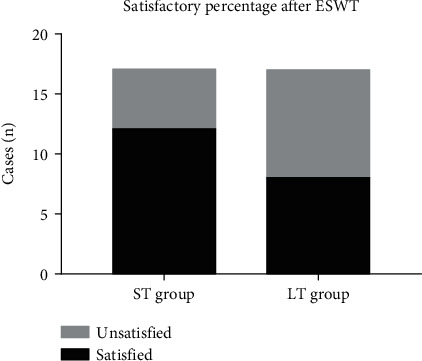
Likert Satisfaction percentage after ESWT.

At the 3rd month after the first ESWT, the satisfaction rate was compared between two groups (Figure 4).

Three months after the first session of ESWT, satisfaction rate of ST group got to 70.6% (12/17), which is higher than that of LT group (47.1%, 8/17). Although the difference did not reach, but is very close to significant level (*p* = 0.163).

### 3.1. Adverse Events

In most of our cases, ecchymosis and numbness were observed and relieved later without any interventions. No bleeding or tendon rupture was reported.

## 4. Discussion

Our results show that ESWT could effectively relieve pain and improve the function of hind foot in CAT patients. Especially, on the aspect of functional improvement, such positive effect is more profound in short course cases. In fact, not only the pathogenesis of CAT is not fully understood [11] but also the therapeutic mechanism of ESWT related to CAT remains not well elucidated [3]. Therefore, better results achieved in short course patients might be closely involved with its pathogenesis and therapeutic mechanism and are worth to be explored.

Epidemiologically, in term of gender tendency, some authors believe that CAT influences more male patients than the female [12], which possibly be related to more frequent physical activities in male exerciser. In fact, a slightly higher incidence of female patients, about 53-59% [13–19], was reported in the majority of literatures, even as high as 77.4% or 81.8% [11, 20] was mentioned. In our study, the general rate of female patient is 53.3% (35/66) and slightly higher than male, which is consist with most of articles. Taylor et al. [21] recruited only refractory CAT patients with average course from 24 to 42 months in their trial. He reported a low prevalence rate in female with longer persistence of symptom. But the rate of female patient with long course is significantly higher than those with short course in our study, which is not only contrary to Taylor's result but also lead to biased character at baseline. Therefore, we believe that if there is any gender tendency in the occurrence of Achilles tendinopathy is still an epidemiological question needs to be explored.

The other bias factor at baseline is BMI. To date, the threshold for overweight in the Chinese population is still 24, which reflects the background of nonuniversality of excessive body weight in China. However, with the change of diet pattern, BMI tends to gradually increase in the Chinese population. In a cross-sectional observational study with large samples, Zhang et al. [22] reported BMI had reached 25.6 ± 3.1 in Chinese male and 23.4 ± 3.2 in the female. Similarly, in our long-term group, BMI was 24.11 ± 2.25, not only greater than in short-term group (23.26 ± 2.15) but also beyond the Chinese threshold of overweight. Although, there is point of view proposes that weight is also an intrinsic fact responsible for CAT [6]; in fact, few literatures focused on BMI level in patients with CAT. In their trials of Achilles tendinopathy, Pinitkwamdee et al. [11] and Mansur et al. [23] described BMI could be as high as 28.2 and 29.2, almost reach the obese standard in the western. Etiologically, developing of CAT possibly because of ankle articular overuse and accumulation of minimal trauma on it [24]. Overweight will cause increased and persisting stress on Achilles's tendon through daily activities; likewise, it could be a possible reason of biased BMI in our long-term group as well.

In the view of therapeutic mechanism, via mechanic stimulation, ESWT could promote the expression of inflammation factors, enhance the proliferation of tenocyte and synthesis of collagen, in turn, repair damaged tendinous tissue and improve the function of Achilles's tendon [25]. Meanwhile, the shock wave could also benefit on the reduction of local substance P [26], destruction of unmyelinated nerve fibres [27], consequently relieve the pain result from CAT. Therefore, pain relief and functional improvement are the most important therapeutic effects. In a systematic review by Al-Abbad and Simon [3], the efficiency of ESWT in pain relief had been consistently proven in 4 trails, and so as functional improvement in 5 trails. In our study, three months after the first shock wave therapy, both AOFAS and VAS scores were improved significantly, which is coincident with most of literatures. Njawaya et al. [19] reported that, with or without guidance of ultrasound, 3 months after ESWT, the converted VAS score is around 2 points, which is very close to our general VAS score (1.92 ± 1.10) measured at corresponding PIM. A placebo-controlled, prospective research from Rasmussen et al. [18] shows ESWT could raise AOFAS to 81.0 at the 3rd month after treatment. Our result manifests an average AOFAS score of 80.49 ± 10.4 in general, which is very similar. Therefore, with our retrospective data, we once more proved the therapeutic effects of ESWT on patients with chronic Achilles tendinopathy overall; no matter the disease course is long or short.

Although, not all the studies took duration of symptom as baseline parameters, but most of authors chose symptom lasting more than 6 months as inclusion criteria, which in turn, make most of patients treated with ESWT, is actually with long disease course. In Taylor et al.'s [21] report, both insertion and noninsertion groups were of long course. 16 weeks after shock wave therapy, the activity VAS score reached only 4.4 and 4 in their groups, respectively. The average course of CAT is 18.23 months in the series recruited by Wheeler et al. [17], and he described the VAS scored only 4.35 at PIM3. In addition, Notarnicola et al. [14] enrolled patients with persisting symptom more than 6 months; 2 months after ESWT, VAS is still 5.4 ± 2.7. On the contrary, Pinitkwamdee et al. [11] took a patient with average disease course of 4.5 months for observation; 16 weeks after ESWT, VAS scores were reduced to 3.00 ± 2.15. Intergroup data were compared after PSM in our study, though VAS score is lower in ST group, but difference is insignificant. The tendency regarding the relationship between longer course and insufficient relief of pain demonstrated in above-listed literatures is not consistently discovered by our study. Leong et al. [28] treated Achilles's tendon of health adults with ESWT, and results showed shock wave could only enhance immediate pressure pain threshold, which restored to normal only 3 hours after intervention. His evidence suggests analgesic effect of ESWT is temporal and transient. Essentially, the quickness of pain relief depends on the speed of neural self-repair. In general, the length of course is a proxy for refractory of certain disease, whether there is a special neurological mechanism underlying the phenomena, in which the pain caused by CAT is hard to be alleviated by short-term shock wave, is a problem need to be investigated in experiment research in the future.

AOFAS is a valuable indicator reflects the function of hind foot, which was often adopted as an outcome parameter in literatures. In Carulli et al.'s [16] series, the average length of disease term is 6.7 months, which is not so prominent in duration. But 6 months after ESWT, AOFAS scores were only improved to 77 ± 2.4. Notranicola et al. [14] recruited patients with symptom duration over 6 months, and 2 months after shock wave therapy, AOFAS raised to only 73 ± 22.9. On the contrary, Vahdatpour et al. [20] observed patients of 4.5 months in disease duration; AOFAS reached as high as 85.85 ± 7.8 at 16 weeks after ESWT. Similarly, Pavone et al. [29] enrolled patients with disease course over 3 months for research, combined with eccentric exercise, AOFAS scores were achieved as 85.2 ± 4.1. As to our ST group, 3 months after the first ESWT, the score of AOFAS went up to 85.08 ± 9.83, which is very similar to the value described by latter two authors, and significantly better than LT group (76.76 ± 9.85). In scoring system of AOFAS, 40 points stand for pain; in other words, only the remaining 50 points of function explain all the changes of AOFAS. In a sense, instead of analgesic effect, ESWT is more powerful in function improvement. The shock wave could transform mechanical stimuli into biological signal which upregulates proliferation of fibroblast, increases genetic expression of TGF-beta1 (transforming growth factor beta-1), collagen I and III, consequently promotes the release of TGF-beta1, synthesis of collagen, and enhances the restoration of tendon as result [30]. Tendon repair is a gradual healing process obviously associated with severity of tissue damage. In most situations, patients with shorter duration are mild in severity, thus are more prone to be benefited by ESWT.

This study has several limitations. As a review study adopting statistical method of propensity score match, theoretically, it has similar evidential value as in prospective studies, but the research is fundamentally retrospective, bias factors might be missed during data collection, will inevitably never be balanced by PSM at baseline, thus limit the power of our study. Secondly, samples are small in our study, some were further lost during match process. Insufficient quantities in sample probably lead to biased conclusion. Therefore, we believe it will be more persuasive to validate our conclusion in prospective, randomized, controlled trials with large sample.

In conclusion, our retrospective study found ESWT could effectively relieve pain and improve impaired function caused by chronic Achilles's tendonipathy. It offers better functional amelioration of hind foot in the patients with short disease course.

## Figures and Tables

**Table 1:** 6-points Likert satisfaction score.

Level of improvement	
Completely recovered	1
Much improved	2
Somewhat improved	3
Hardly improved	4
Not improved	5
Worse	6

**Table 2:** Baseline features in patients with chronic Achilles tendinopathy before PSM.

Characteristics	ST group (*n* = 45)	LT group (*n* = 21)	t (*χ*^2^)	*p*
Gender [*n* (%)]				
Male	25 (55.6)	6 (29.6)	4.186	0.041^∗^
Female	20 (44.4)	15 (71.4)		
Sports [*n* (%)]				
Yes	28 (62.2)	10 (47.6)	1.25	0.264
No	17 (37.8)	11 (52.4)		
Sides [*n* (%)]				
Superiority	26 (57.8)	12 (57.1)	0.002	0.961
Nonsuperiority	19 (42.2)	9 (42.9)		
Type [*n* (%)]				
Insertion	17 (37.8)	7 (33.3)	0.122	0.727
Noninsertion	28 (62.2)	14 (66.7)		
BMI	23.26 ± 2.15	24.63 ± 2.41	-2.317	0.024^∗^
Age (*y*)	56.77 ± 8.68	60.80 ± 8.74	-1.754	0.084
AOFAS	63.88 ± 10.44	63.06 ± 11.52	0.288	0.774
VAS	6.03 ± 1.09	5.61 ± 1.13	1.431	0.157

^∗^Compared with ST group, the difference is significant, *p* < 0.05.

**Table 3:** Baseline features and outcome parameters in patients with chronic Achilles tendinopathy after PSM.

Characteristics	ST group (*n* = 17)	LT group (*n* = 17)	t (*χ*^2^)	*p*
Gender [*n* (%)]				
Male	6 (35.3)	4 (23.5)	0.567	0.452
Female	11 (64.7)	13 (76.5)		
Sports [*n* (%)]				
Yes	12 (70.6)	8 (47.1)	1.943	0.163
No	5 (29.4)	9 (52.9)		
Sides [*n* (%)]				
Superiority	10 (58.8)	10 (58.8)	0	1
Nonsuperiority	7 (41.2)	7 (41.2)		
Type [*n* (%)]				
Insertion	6 (35.3)	7 (41.2)	0.124542	0.724
Noninsertion	11 (64.7)	10 (58.8)		
BMI	23.96 ± 1.93	24.11 ± 2.25	0.215	0.831
Age (*y*)	59.91 ± 9.62	61.34 ± 9.33	0.441	0.662
Preintervention				
AOFAS	65.43 ± 10.05	62.80 ± 11.90	0.695	0.492
VAS	5.92 ± 1.12	5.87 ± 1.10	0.127	0.9
Postintervention				
AOFAS	85.08 ± 9.83^∆∆^	76.76 ± 9.85^∆∆^	2.464	0.019^∗^^∆^
VAS	1.96 ± 0.98^∆∆^	2.24 ± 1.29^∆∆^	0.703	0.487
Sat. rate [*n* (%)]	12 (70.6)	8(47.1)	1.943	0.163

^∗^Compared with ST group in same period, the difference is significant, and *p* < 0.05; ^∆∆^compared with preintervention in same group, the difference is significant, and *p* < 0.01.

## Data Availability

The data used to support the findings of this study are available from the corresponding author upon request.

## Abbreviations

BMI: Body mass index
CAT: Chronic Achilles tendinopathy
ESWT: Extracorporeal shockwave therapy
HA: Hyaluronan
PIM: Postinterventional month
PSM: Propensity scores match
TGF-beta1: Transforming growth factor beta-1
VAS: Visual Analogue Scale.
